# Natural History, Microbes and Sequences: Shouldn't We Look Back Again to Organisms?

**DOI:** 10.1371/journal.pone.0021334

**Published:** 2011-08-16

**Authors:** Antonio Lazcano

**Affiliations:** Facultad de Ciencias, Universidad Nacional Autónoma de Mexico, Cd. Universitaria, México; American Museum of Natural History, United States of America

## Abstract

The discussion on the existence of prokaryotic species is reviewed. The demonstration that several different mechanisms of genetic exchange and recombination exist has led some to a radical rejection of the possibility of bacterial species and, in general, the applicability of traditional classification categories to the prokaryotic domains. However, in spite of intense gene traffic, prokaryotic groups are not continuously variable but form discrete clusters of phenotypically coherent, well-defined, diagnosable groups of individual organisms. Molecularization of life sciences has led to biased approaches to the issue of the origins of biodiversity, which has resulted in the increasingly extended tendency to emphasize genes and sequences and not give proper attention to organismal biology. As argued here, molecular and organismal approaches that should be seen as complementary and not opposed views of biology.

## Introduction

Although the actual number of biological species remains undetermined, all estimates suggest very high figures, which would reach staggering levels if the myriads of microbial groups could be calculated. There are additional complications. Although many biologists loyally adhere to traditional definitions of species that are clearly valid for animal and plant clades, the alarming flow of medical reports of antibiotic resistance in many microbial pathogens, and the availability of many fully sequenced genomes have made many aware of the extraordinary porosity of the taxonomic barriers separating prokaryotic taxa. As discussed below, this has led some to a radical rejection of the concept of bacterial species and, in fact, to a renewed discussion of the applicability of traditional classification categories to the prokaryotic domains.

## Sex in the wild (and human-mediated breeding in captivity)

It is unfortunate that nowadays many life scientists and students think of taxonomy as a name-revering discipline obsessed with etymologies, and of Linneus as the mere inventor of binomial nomenclature and not as the founder of modern biological systematics. Although he was critized by some of his contemporaries as being excessively wordy, the first edition of his *Systema Naturae* has in fact only a dozen large pages of text, followed by well-designed double-page spreads in which Linneus presented a meticulous, innovative classification of the Mineral, the Vegetable and the Animal kingdoms [1].

Described by a contemporary English publication as “the greatest Botanist that the world ever did or will probably ever will know”, Linneus deep understanding of the natural history of plants led him to recognize the usefulness of sexual reproduction as the basis of his classification schemes. Perhaps not surprisingly, the criteria he employed lead some of his more pious and unworldly colleagues to reject his approach and to accuse him of creating an immoral sexual system of classification –charges that were so virulent, that long before political correctness became fashionable, he had to face allegations of “loathsome harlotry”. He couldn't care less and, being a staunch believer, remained convinced not only of the usefulness of the criteria he had developed, but also that the hierarchical patterns of resemblances and differences between animal and plant species and their grouping into higher taxa on the basis of shared similarities revealed not a process of change but their ultimate divine origin [2], [3]. Throughout his life Linneus maintained that biological species are perfectly defined, real groupings consisting of individuals bound together by reproduction, in which progeny resembles their progenitors.

Such criteria are still used by many, but the definition of a species remains a problem. The problem has not faded away but keeps bouncing back and remains a highly contentious issue, as shown by the manifold (and sometimes opposing) concepts of species that are used in different areas of biology and in everyday life in different professional circles and different societies [4]. The issue is complicated by the many demonstrations of the interbreeding promiscuity of species in both plants and animals. Plant hybridization is a well-documented phenomenon that actually led Linneus during his late years to acknowledge the possibility of the emergence of new species. Reports of interbreeding between related animal species also demonstrate that although ecological distribution, anatomical traits and physiological differences can impose major barriers to crossbreeding, absolute reproductive isolation may not be a reality among different species.

The list of organisms resulting from such taxonomic promiscuity is small, but this may be an artifact. It includes tigons, for instance, which are hybrids between a male tiger and a female lion (which would group the resulting organism with lions if the mitochondrial cytochrome *c* oxidase subunit used in barcoding would be employed), as well as many other chimeras like zebroids and zonkeys. It is true that these and other cross-breeds have been born because of human intervention, but mitochondrial DNA analysis has shown that similar interspecies crossbreeding events have occurred between grizzly and polar bears and, more recently, that male wolves and female coyotes mate in the wild [5]. Such hybridization processes probably represent an evolutionary mechanism generating adaptative variation in biological populations in the wild.

The contemporary listing of the known mechanism of genetic exchange and recombination between different species would have horrified Linneus' most prudish detractors. Many varieties of sexual genetic recombination have been described, including the mating habits of fungal strains, nuclear fusion in yeast and other ascomycetes, vegetative fusion in algae, conjugation in amoeba and paramecia and, among prokaryotes, an extended network of viral crossover and plasmid and phage-mediated lateral gene transfer [3]. Existing life forms may not be mere nodes in a genetic reticulated network that can overcome all taxonomic barriers, but the available evidence shows that interspecific chastity is not as strict as some would like to assume.

## A rose is a rose is a rose –but what about lateral gene transfer and symbiotic associations?

Antagonistic taxonomies have coexisted more or less peacefully along the history of biology. However, the definition of prokaryotic species is a highly contentious issue, and has led in some cases to the overall rejection of the applicability of Linnean taxonomic categories to the Bacteria and Archea. Polyphasic definitions of bacterial species depend on quantitative 16S rRNA divergence values [6]. By definition, individual strains of a bacterial species can differ by up to 30% in terms of genetic sequence, i.e., assignment of isolates to species is based on measures of phenotypic or genome similarity. The usefulness of this approach is well established, but cladistic analyses of rRNA sequences do not necessarily guarantee by themselves a proper delineation of prokaryotic species [7], and gene phylogenies that conflict with canonical rRNA trees do suggest extensive traffic of genes and sequences (which may have been much more intense during the early history of the biosphere) that connect otherwise separate groups of prokaryotes [8].

Discord confuses what prokaryotic species mean or should mean, and microbiologists keep struggling to find a definition. One radical choice is to do with the concept altogether. However, although lateral gene transfer is a major obstacle to establishing clear demarcation lines between prokaryotes, the microbial world is not an evolutionary continuum that seamlessly joins diverse groups. Biologically or ecologically meaningful sequence clusters are recognizable, which implies that in spite of intense gene traffic, prokaryotic groups are not continuously variable but form discrete clusters of phenotypically coherent, well-defined, diagnosably groups of individual organisms. In spite of the still undetermined levels of widespread lateral gene transfer, such “lumpy” structures are biologically or ecologically meaningful sequence clusters that demonstrate that the archaeal and bacterial genome sequence spaces are somewhat less astringent than those of eukaryotes. The empirical recognition of different prokaryotic groups demonstrates that although their genomic identity is not as strictly defined as in animals, selection maintains them as somewhat hazy clusters in local fitness peaks forming clumpy landscapes.

There are other somewhat less intimate ways in which different species can associate. The recent report of the complete sequence of the giant panda genome concluded that “[…] our analysis of genes potentially involved in the evolution of the panda's reliance on bamboo in its diet showed that the panda seems to have maintained the genetic requirements for being purely carnivorous even though its diet is primarily herbivorous. Furthermore, given our finding that some of the genes necessary for complete digestion of bamboo are missing from its genome, investigation of panda's gut microbiome may be important for understanding its unusual dietary restrictions.” [9].

The bottom line is, of course, that the availability of a completely sequenced genome is not enough to understand the biology of the giant panda or, for that matter, of all animals, including us. Current estimates suggest that we host around one thousand prokaryotic species in our gut, as well a still undetermined numbers of associated microbes in the external and internal body surfaces [10], [11]. No species lives in blissful isolation from other taxa. This is particularly true of prokaryotes, which are now recognized as essential symbiotic partners in the development and extensive distribution of plants and animals. Proper description of the genetic inventory of organisms is a dauntingly complex task, but the overall understanding of *Elysia viridis* and other mollusks whose semi-autotrophic lifestyle is strongly dependent on secondarily acquired chloroplasts [12] which are inherited, like the panda microbiome, by non-Mendelian mechanisms, demonstrates that the detailed description of a complete genome does not suffice to describe in full the biology of an organism.

## So close to the sequences, so far from the phenotype

It is somewhat unfortunate that not all are aware of the limits of genetic reductionism in our description and understanding of biological diversity and evolution. As shown by the extraordinary achievements of molecular biology, contemporary life sciences have achieved unsurpassed progress through methodological Cartesian reductionism. Unfortunately, these achievements have gone hand in hand with the failure to recognize that the molecular and genetic components of a living system do not exist in isolation but come into being as a function of their context. Since the teaching of the natural history of organisms is seen by many individuals, institutions and funding agencies as *démodé*, throughout the world we are failing to provide students with a balanced view of molecular and organismal approaches that should be seen as complementary and not opposed.

Molecular approaches to taxonomic and evolutionary questions are not new. In 1904 the American-born British naturalist and physician George H. F. Nuttall published a volume summarizing the results of his detailed comparison of blood proteins used to reconstruct the evolutionary relationships of animals. “In the absence of paleontological evidence”, wrote Nuttall, “the question of the interrelation-ship among animals is based upon similarities of structure in existing forms. In judging these similarities, the subjective element may largely enter, in evidence of which we need to look at the history of the classification of the Primates” [13]. Such a subjective element, Nuttall argued, could be successfully overcome by constructing a phylogeny based not on form but on the immunological reactions of blood-related proteins [14].

However, as time went by some unsuspected conflicts began to develop, that signaled a growing cultural split between the newborn geneticist guild and the old school of naturalists. As Mayr wrote in 1988, “the emphasis on the role of diversity in evolution was stressed by naturalists from Darwin on, but was almost totally ignored by the Fisherian; the naturalists, for their part, rejected the beanbag genetics of the reductionists and the post-synthesis period continued their holistic tradition of emphasizing the individual as the target of selection” [15].

We are still suffering the negative effects of such divorce. The pioneering efforts of Kluyver, Florkin and few others in comparative biochemistry reflected the molecularization of systematics and evolutionary biology, but they remained in the outskirts of mainstream research. Molecular biologists did not embrace these views, and during several decades evolutionary approaches were frequently dismissed as little more than useless speculation. This skeptical attitude started to change with the awareness that genes and proteins are rich historical documents from which a wealth of evolutionary information can be retrieved [16]. A major change occurred when the evolutionary comparison of small ribosomal RNA (rRNA) led to the description of most of the newly classified Bacteria and Archaea species [17], [18]. All of a sudden, the discovery that prokaryotes were divided into what we call now Bacteria and Archaea led molecular biology to accept that the existence of important differences among the three domains of life required looking beyond the way in which few model organisms like *Escherichia coli*, *Saccharomyces cerevisae* and *Arabidopsis thaliana* store, replicate and express their genetic information [19].

As time went by, however, sequences of DNA and proteins became the focus of investigation. The replacement of phenotypic delimitation of species with one based on DNA comparisons has taken place at a quick pace [20]. The power of DNA technology is beyond dispute, but these two different perspectives are premised on a meaningless opposition. Comparative phylogenomics requires not only the development of less-expensive, more rapid genome sequencing techniques, more powerful computer algorithms for constructing phylogenetic trees and better organized databases, but also the critical awareness of its non-stated reductionist assumptions and more precise definitions of its conceptual framework. Genome trees [21], barcoding [22], and DNA taxonomy [20] are useful, ingenious outcomes of the process of molecularization of biology that help to describe biodiversity, but do not explain it. Other recent developments, including phylogenomics, require not only a substantial knowledge and understanding of phylogenetic analysis and computational skills to handle the large-scale data involved, but also the recognition of the usefulness of the phenotype in understanding the ultimate evolutionary causes underlying past and present biological diversity [23].

## Conclusions

The development of efficient sequencing techniques, combined with the simultaneous and independent blossoming of computer science has led not only to an explosive growth of databases and new sophisticated tools available for their exploitation, but also to the recognition that different macromolecules may be uniquely suited as molecular chronometers in the construction of increasingly complete phylogenies. There has been a flood of nucleic acid sequence information, bioinformatic tools and phylogenetic inference methods in scientific publications, public domain databases, and the worldwide web space, but they need to be complemented with a proper understanding of the phenotypic traits and the natural history of organisms. Contemporary biology tends to forget that genomes and phenotypes are so deeply intertwined that attempts to partition the causal interdependence is simply meaningless.

In 1975 the distinguished ecologist G. E. Hutchinson stated that “…many ecologists of the present generation have great ability to handle the mathematical basis of the subject. Modern biological education, however, may let us down as ecologists if it does not insist, and it still shows to few signs of insistence, that a wide and quite deep understanding of organisms, past and present, is as basic a requirement for anything else in ecological education. It may be best self-taught, but how is this difficult process made harder by a misplaced emphasis on a quite specious modernity” [24]. Substitute “molecular biology or comparative genomics” where Hutchinson wrote “mathematics”, and his statement reflects the current situation. However fruitful, such approaches have all the demerits of a reduccionist, one-trait approach to our understanding of the mechanism underlying biological diversity.

As summarized elsewhere [14], we can overcome such limitations in several ways, some of which are part of intellectual traditions deeply rooted in comparative biology. As Georges Cuvier contended in his 1805 extensive *Leçons d'anatomie comparée*, the appearance of the whole skeleton can be deduced up to a certain point by examination of a single bone. The success that Cuvier had in such anatomical reconstruction is legendary, and was based not only in his unsurpassed knowledge and intuition, but also in what he termed the “correlation of parts”, i.e., the full recognition of a functional coordination of the body of a given animal [1]. Such correlation of parts is not restricted to bones and muscles and, in contrast to Cuvier, we should not put aside the evolutionary history of biological systems. With very few exceptions, however, molecular phylogeny has rarely been used to attempt a truly integrative analysis of complete character complexes.

It is equally important to reevaluate the usefulness of a phenotype-based organismal approach in biodiversity and evolutionary issues. Part of the solution depends in educating the new generations of scientists the true value of natural history, and not to discard a valuable and central concept in biology. This should be read as a plea for a more integrative approach in the study of biodiversity and its underlying causes that goes beyond sequence analysis. Resources are flooding in an asymmetric way to life sciences, bringing with them a biased recognition of the different approaches to our understanding of biological phenomena. Organismal biology needs to be supported, renewed and recognized as a central component, both in education and research, of contemporary life sciences. It not too late for doing so, but time is running fast.
